# Comparative evaluation of platelet rich plasma in socket healing and bone regeneration after surgical removal of impacted mandibular third molars

**DOI:** 10.15171/joddd.2018.024

**Published:** 2018-09-18

**Authors:** Ravi Bhujbal, Neelima A Malik, Nilesh Kumar, Suresh KV, Mushtaq I Parkar, Jeevan MB

**Affiliations:** ^1^Department of Oral and Maxillofacial Surgery, Nanded Rural Dental College & Research Center, Vishnupuri Nanded, Maharashtra, India; ^2^Department of Oral and Maxillofacial Surgery, School of Dental Sciences, Krishna Institute of Medical Sciences Deemed University, Karad, Maharashtra, India; ^3^Faculty of Dentistry, SEGi University, Selangor, Malaysia; ^4^Department of Oral Pathology, Faculty of Dentistry, AMIST University, Kedah Darul Aman, Malaysia

**Keywords:** Extraction, platelet-rich plasma, soft tissue healing, pain, swelling, third molar

## Abstract

***Background.*** Surgical removal of mandibular third molars results in pain, swelling and bony defects, causing prolonged postoperative recovery. The growth factors present in platelet-rich plasma (PRP) can accelerate the healing, thereby shortening postoperative recovery period. This study was undertaken to evaluate the role of PRP in postoperative socket healing, pain, swelling and bone regeneration following surgical removal of impacted mandibular third molars.

***Methods.*** The present case‒control study was conducted on 20 patients with identical bilateral mandibular third molar impaction. PRP was placed randomly on one side of 3rd molar extraction socket and the contralateral side was used as control. Evaluation of soft tissue healing, pain, swelling and radiologic bone density was carried out.

***Results.*** Soft tissue healing was better in the PRP compared to the control site. Immediate postoperative assessment of pain scores showed no significant difference between the two groups (Mann-Whitney U test). On the 7th day, pain scores were lower in case site compared to the control site. Measurement of swelling on the 1st, 3rd and 7th day showed statistically significant differences between the case and control sites (P<0.0001). Postoperative mean bone density at the 3rd and 6th postoperative months was significantly higher in the case site compared to the control site (P=0.00001).

***Conclusion.*** The results showed an improvement in wound healing and swelling and an increase in the bone density at PRP site. The growth factors in PRP would improve the hard and soft tissue healing 3 months after molar surgery.

## Introduction

Healing of surgical extraction site is a highly coordinated sequence, involving growth factors, hormones and cytokines. Platelet-rich plasma** (**PRP) is a derivative of platelets which consists of numerous growth factors (GF) such as, transforming GF-β, vascular endothelial growth factor (VEGF), and epithelial GF, all of which accelerate wound healing.^1,2^ Apart from favoring tissue healing, it also stimulates angiogenesis and decreases pain and swelling.^3-5^ Extraction of mandibular third molar is invariably associated with pain, swelling and osseous defects. The GFs present in PRP can accelerate healing, thereby improving postoperative recovery. Previous studies on PRP mainly focused on enhancing the healing in bone grafts and dental implants. There is a lack of information in the literature regarding the role of PRP in extraction socket healing and bone regeneration in mandibular third molar surgery. In this study, an attempt was made to evaluate the soft tissue healing, pain, swelling and radiological bone density in patients undergoing identical bilateral mandibular third molar surgery.

## Methods

The present case‒control study was conducted in the Department of Oral and Maxillofacial Surgery. The ethical clearance was obtained from the Ethics Committee. A total 20 patients with identical bilateral mandibular third molar impaction were included. One side of the extraction socket was taken as the case site and the contralateral side as the control site in the same patient. All the patients were informed about the nature of the study and written informed consent was obtained before participating in the study. Healthy patients (ASA I) with bilateral identical impacted mandibular third molars, who were willing to return for follow-up visits, were included in the study. The complexity of extraction was assessed by the index described by Pederson.^6^ PRP was placed randomly on one side of 3rd molar extraction socket in the case site and the contralateral site was used as control. Postoperative evaluation of soft tissue healing, pain, swelling and radiologic bone density was carried out.

### Exclusion criteria

- Patients with localized infection in mandibular third molars.
- Immunocompromised status or patients with any systemic disease affecting the healing process.
- Patients with adverse oral habits such as smoking and alcohol.
- Patients with platelet count <150,000/cu.mm with a history of bleeding disorder.

### Preparation of PRP

Ten mL of intravenous blood was drawn from the antecubital region of patients using a flashback blood collection needle and BD vacutainer containing citrate phosphate dextrose adenine solution. The vacutainer containing blood was centrifuged at 1200 rpm for 10 minutes to separate the blood into a lower part containing red blood cells (RBCs) and upper straw-colored plasma. The upper plasma contains low concentration of platelets (platelet-poor plasma; PPP) and relatively higher concentration of platelets in the boundary layer called "buffy coat".

PPP, buffy coat and upper 1 mL RBC layer was collected in a 12-mL borosilicate glass tube and was counter-balanced and centrifuged at 2000 rpm for 10 minutes. The upper half of the supernatant was discarded and the lower half was mixed to yield PRP and transferred into a clean sterile stainless steel bowl and 0.5‒1 mL of 10% calcium chloride was added to the PRP, leading to the formation of PRP gel.

### Surgical extraction of third molar

A conventional inferior alveolar nerve block was given using 2% lignocaine hydrochloride with 1:80,000 adrenaline. A standard classical ward incision was given and mucoperiosteal flap was raised. The bone covering the distobuccal aspect of the impacted tooth was removed using a round bur. The impacted tooth was removed from the socket with the help of dental elevators. The extracted socket was irrigated with 0.5% diluted povidone iodine (BETADINE, G.S. Pharmabutor Pvt Ltd., Uttarakand and distributed by Win-Medicare, New Delhi, India).^6^ This served as a hemostyptic, antiedematous agent and prevented infection at surgical site.

**Figure 1:**
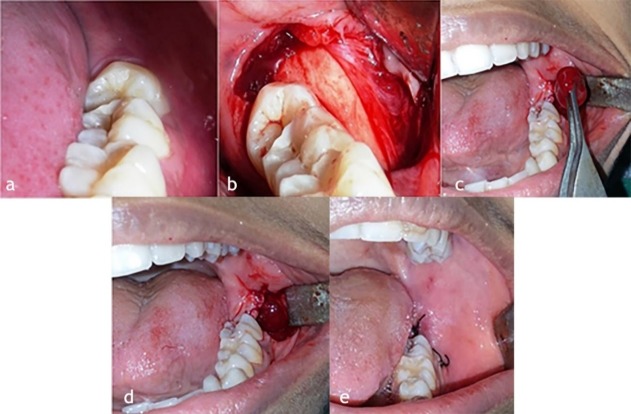
Steps of surgical removal of the left impacted third molar and placement of PRP. (a) Left impacted third molar. (b) Reflection of mucoperiosteal flap. (c) PRP being placed in the third molar socket. (d) PRP in the socket. (e) Wound closed with 3-0 black silk interrupted sutures.

Primary wound closure was carried out using 3-0 black silk by interrupted sutures after PRP placement (Figure 1). Surgical procedures were performed by a single experienced oral and maxillofacial surgeon, who was observer too and the same set of sterilized equipment was used in the all extractions.

The soft tissue healing, pain and swelling were measured on the 1st, 3rd and 7thpostoperative day. Soft tissue healing was assessed in terms of the healing index given by Laundry and Turn Bull (1988). The parameters evaluated included change of tissue color more than 50% gingival red, bleeding on palpation and granulation tissue formation.

Pain assessment was carried out using subjective visual analogue scale (VAS). Pre- and postoperative swelling was assessed by a flexible plastic measuring tape. The landmarks included horizontal dimension of swelling (from the lower attachment of the ear lobe to the corner of the mouth) and vertical dimension of swelling (from angle of mandible to the outer cantus of the eye).

The bone density was assessed using digital panoramic radiograph (OPG) 3and 6 months postoperatively. The mean gray level histogram values of the OPG at extraction socket were obtained through Adobe Photoshop 7.0 software. The difference in mean grey level values at study and control sites were tabulated and compared.

## Results

The present study comprised of 5 male (25%) and 15 female (75%) patients** (**mean age=25.20 and SD=7.19). In the case group site, there were no patients with tissue color more than 50% gingival red, bleeding on palpation or granulation tissue formation. In the control site tissue color more than 50% red was present in two patients on the 3rdday and in one patient on the 7th day. Bleeding on palpation was present in one patient on the first day. Thus soft tissue healing was better in the case site compared to the control site (Table 1).

**Table 1:** Comparison of the study and control groups in terms of the tissue color more than 50% gingival red, bleeding on palpation and granulation tissue

**Group**	**Tissue color more than 50% gingival red**			**Bleeding on palpation**			**Granulation tissue**		
	1st day	3rd day	7th day	1st day	3rd day	7th day	1st day	3rd day	7th day
**Study group**	0	0	0	0	0	0	0	0	0
**Control group**	0	2	1	2	0	0	0	0	0

### Assessment of pain

Postoperative pain was measured using VAS. The pain scores were lower in the case site compared to the control site. However, statistical analysis by Mann-Whitney U test showed no significant difference between immediate postoperative pain and after 1, 3 and 7days between the case and control groups (Table 2).

**Table 2:** Comparison of the study and control groups with respect to VAS scores by Mann-Whitney U test

**Groups**	**Immediate post-operative**		**1st day**		**3rd day**		**7th day**		**Changes from immediate post-operative to**					
									**1st day**		**3rd day**		**7th day**	
	**Mean**	**SD**	**Mean**	**SD**	**Mean**	**SD**	**Mean**	**SD**	**Mean**	**SD**	**Mean**	**SD**	**Mean**	**SD**
**Study**	2.0	0.9	1.6	0.6	2.0	0.7	0.0	0.0	0.4	1.0	-0.1	1.1	2.0	0.9
**Control**	2.3	0.9	2.1	0.6	2.4	0.9	0.1	0.2	0.3	0.9	-0.1	1.4	2.3	1.0
**Z-value**	-1.1632		-1.9882		-1.3525		-0.2705		-0.1217		-0.0271		-1.0685	
**P-value**	0.2448		0.0468		0.1762		0.7868		0.9031		0.9784		0.2853	

*P<0.05, # applied Wilcoxon matched pairs test

### Assessment of swelling

Vertical and horizontal dimensions of the swelling were measured on the 1st, 3rd and 7th days. There were significant decreases swelling on the case side on the 1st (P=0.0059) and 3rd days (P=0.0001). However, on the 7th day, there was no statistically significant difference between the case and control sites (P=1.00) (Tables 3 and 4).

**Table 3:** Comparison of the study and control groups with respect to vertical swelling scores (in cm) by unpaired t-test

**Groups**	**Immediate pre-operative**		**1st day**		**3rd day**		**7th day**		**Changes from immediate pre-operative to**					
									**1st day**		**3rd day**		**7th day**	
	**Mean**	**SD**	**Mean**	**SD**	**Mean**	**SD**	**Mean**	**SD**	**Mean**	**SD**	**Mean**	**SD**	**Mean**	**SD**
**Study**	9.65	0.99	9.81	1.00	9.84	1.00	9.64	0.99	0.16	0.07	0.19	0.07	-0.01	0.04
**Control**	9.65	0.99	9.88	0.98	9.96	1.01	9.64	0.99	0.23	0.09	0.31	0.09	-0.01	0.04
**t-value**	0.0000		-0.2402		-0.3784		0.0000		-2.9153		-4.4607		0.0000	
**P-value**	1.0000		0.8115		0.7072		1.0000		0.0059*		0.0001*		1.0000	

*P<0.05, # applied paired t-test

**Table 4:** Comparison of the study and control groups with respect to horizontal swelling scores (in cm) by unpaired t-test

**Groups**	**Immediate pre-operative**		**1st day**		**3rd day**		**7th day**		**Changes from immediate pre-operative to**					
									**1st day**		**3rd day**		**7th day**	
	**Mean**	**SD**	**Mean**	**SD**	**Mean**	**SD**	**Mean**	**SD**	**Mean**	**SD**	**Mean**	**SD**	**Mean**	**SD**
**Study**	11.19	0.47	11.34	0.44	11.38	0.44	11.19	0.47	0.16	0.08	0.20	0.08	0.00	0.00
**Control**	11.19	0.47	11.41	0.45	11.48	0.43	11.19	0.47	0.22	0.12	0.30	0.11	0.00	0.00
**t-value**	0.0000		-0.4615		-0.7253		0.0000		-1.9997		-3.3481		--	
**P-value**	1.0000		0.6471		0.4727		1.0000		0.0527		0.0018*		--	

*P<0.05, # applied paired t-test

### Radiologic assessment

Mean bone density scores at 3rdmonth in case and control sites were 131.24 and 131.21, respectively (P<0.01); at the 6th month these scores were 135.67 and 133.80 in the case and control sites, respectively (P<0.00001). There were significant differences in bone density between the case and control sites 3 and 6 months postoperatively (Table 5).

**Table 5:** Comparison of the study and control groups with respect to bone density scores by unpaired t-test

**Groups**	**3rd day**		**1 month**		**3 months**		**6 months**		**Changes from 3rd day to**					
									**1 month**		**3 months**		**6 months**	
	**Mean**	**SD**	**Mean**	**SD**	**Mean**	**SD**	**Mean**	**SD**	**Mean**	**SD**	**Mean**	**SD**	**Mean**	**SD**
**Study**	126.06	3.95	128.96	4.12	131.24	4.33	135.67	4.04	2.90	0.90	5.19	1.33	9.61	1.45
**Control**	127.18	4.44	129.03	4.62	131.21	4.78	133.80	4.97	1.86	1.30	4.03	1.49	6.62	2.34
**t-value**	-0.8444		-0.0549		0.0232		1.3057		2.9572		2.5903		4.8573	
**P-value**	0.4037		0.9565		0.9816		0.1995		0.0053*		0.0135*		0.00001*	

*P<0.05, # applied paired t-test

## Discussion

Platelet-rich plasma contains bioactive proteins and growth factors that stimulate and accelerate wound healing process. It also provides a matrix formed by a high concentration of viable platelets, providing a scaffold needed for cellular movement and proliferation.^7^

PRP gel is a product of platelet with thrombin and calcium chloride, which was initially used as a soft tissue healing agent.^8^ In the present study, soft tissue healing was assessed in terms of the healing index given by Laundry & Turn Bull (1988),^8^ on the 1st, 3rd and 7th postoperative days. Soft tissue healing was found to be better at PRP gel site compared to the control site, consistent with previous the studies by Anitua et al (1999),^3^ Sammartino et al (2005),^9^ Vivek and Sripati Rao (2009),^8^ Alissa et al (2010),^10^ Mozzati et al (2010)^11^ and Ogundipe et al (2011).^12^ It might be concluded that PRP has a facilitative role in soft tissue wound healing.

Postoperative pain was assessed by VAS on immediate 1st, 3rdand 7th postoperative days. Less pain was present postoperatively at PRP compared to the control site. However, the difference was not statistically significant. Similar findings were noted by studies conducted by Mancuso et al (2003),^13^ Vivek et al (2009),^8^ Gawande et al (2009,)^14^ Alissa et al (2010),^10^ Mozzati et al (2010)^11^ and Ogundipe et al (2011).^12^ However, a study by James et al (2010)^15^ could not find any significant difference in pain between the PRP and non-PRP groups following third molar surgery.

There was relatively less postoperative swelling at the PRP site compared to the control site. However, no significant difference was seen on the 7th day between the control and case sites. Mozzati et al (2010),^11^ Alissa et al (2010)^10^ and Gawande et al (2009)^14^ reported a decrease in facial swelling following application of PRP gel to extraction sockets as compared to the control side.

The bone healing was more favorable in the PRP site compared to the control site. A significant increase in bone density was seen on the PRP site 3 and 6 months postoperatively. Anitua et al (1999),^3^ Mancuso et al (2003),^13^ Goto et al (2008),^16^ Alissa et al (2010),^10^ Ogundipe et al (2010),^12^ Del et al (2011)^17^ and Antonello (2013)^18^ reported better epithelialization, more mature bone and better organized trabeculae than controls, and accelerated bone formation postoperatively in the PRP group compared to the control group. However, Earl et al (2004)^19^ did not report substantial bone formation when PRP was applied.

## Conclusion

The results of the present study showed improvements in wound healing and an increase in bone density in the PRP site compared to the control site. Although immediate postoperative pain and swelling was lower in the PRP site, this was not statistically significant. Radiographically, bone density was higher in the PRP site at the 6-th month follow-up, which signifies the importance of PRP as a valid method in accelerating wound healing in patients undergoing 3rd molar surgery.

## Acknowledgements

None.

## Authors’ contributions

RB, NAM and SKV were involved in designing the study and collection of data. The rest of the authors were involved in drafting and final approval of the manuscript.

## Funding

The authors report no funding for this study.

## Competing interests

The authors declare no competing interests with regards to the authorship and/or publication of this article.

## Ethics approval and Patient consent

The ethical clearance was obtained from the institutional ethics committee. All the patients involved in the study gave written consent for participation and publication of the research data.
